# Editorial: Molecular diagnostics of pediatric cancer, volume II

**DOI:** 10.3389/fonc.2024.1493791

**Published:** 2024-09-27

**Authors:** Jing He, Yizhuo Zhang, Hua Tan, Jochen Rössler, Jinhong Zhu

**Affiliations:** ^1^ Department of Pediatric Surgery, Guangzhou Institute of Pediatrics, Guangdong Provincial Key Laboratory of Research in Structural Birth Defect Disease, Guangzhou Women and Children’s Medical Center, Guangzhou Medical University, Guangzhou, Guangdong, China; ^2^ Department of Pediatric Oncology, State Key Laboratory of Oncology in South China, Collaborative Innovation Center for Cancer Medicine, Sun Yat-Sen University Cancer Center, Guangzhou, Guangdong, China; ^3^ School of Biomedical Informatics, The University of Texas Health Science Center at Houston, Houston, TX, United States; ^4^ Division of Pediatric Hematology and Oncology, Department of Pediatrics, Inselspital, Bern University Hospital, University of Bern, Bern, Switzerland; ^5^ Department of Clinical Laboratory, Biobank, Harbin Medical University Cancer Hospital, Harbin, Heilongjiang, China

**Keywords:** pediatric cancer, prognostic, biomarker, molecular target, therapeutics

Pediatric tumors refer to various types of malignant tumors occurring in children and adolescents. Common pediatric tumors include brain and central nervous system tumors, lymphomas, neuroblastoma, Wilms tumor, osteosarcoma, rhabdomyosarcoma, and leukemia. Unlike adult cancers, pediatric tumors often exhibit unique genetic profiles and biological behaviors, necessitating age-specific diagnostic and therapeutic approaches (1). Molecular diagnostics have revolutionized pediatric cancer care, shifting diagnosis and treatment from traditional histological and clinical methods to a deeper understanding of the genetic basis of these malignancies. Innovations such as next-generation sequencing (NGS) and liquid biopsy techniques consequently expanded our ability to detect genetic mutations, chromosomal abnormalities, and tumor-specific biomarkers with unprecedented accuracy and sensitivity. However, molecular diagnostics in pediatric cancer still face significant challenges. First, the rarity of certain pediatric cancers limits the availability of large, statistically powerful cohorts needed for comprehensive molecular studies. Second, the diversity and complexity of pediatric tumors further complicate the diagnostic landscape. This complexity is particularly evident in neuroblastoma, where diverse genetic alterations and varying disease stages demand a more meticulous understanding of molecular underpinnings to improve diagnostic accuracy and treatment outcomes (2). This Research Topic brings together nine insightful studies that collectively expand our understanding of molecular diagnostics in pediatric cancer. The Research Topic cover a broad spectrum of pediatric malignancies and molecular mechanisms, spotlighting the intricate interplay between genetic and molecular factors in tumorigenesis and disease progression.

By retrospectively analyzing 21 retinoblastoma (RB) specimens, Al-Ghazzawi et al. demonstrated the pivotal role of growth factors in driving tumor development in high-risk RB, including platelet-derived growth factor (PDGF), nerve growth factor (NGF), and epidermal growth factor (EGF). Kaposiform Hemangioendothelioma (KHE), a rare, life-threatening, regional vascular tumor, primarily affects infants and young children. The present case report identified a *PIK3CA* mosaic pathogenic variants (c.685delA and p.Thr229fs) in KHE and offered a successful example of using sirolimus (also known as rapamycin) for KHE with *PIK3CA* mutations (Wang et al.). These findings align with the conception that KHE may belong to the PIK3CA-related overgrowth spectrum (PROS), a heterogeneous group of disorders resulting from activating variants of the PIK3CA gene (3, 4).

Neuroblastoma, a recurrent theme in this Research Topic, is explored through several perspectives: the potential of circular RNAs (circRNAs) as diagnostic biomarkers, PET/CT imaging in relation to *MYCN* gene status, mitochondrial gene expression, differentially expressed genes (DEGs) between neuroblastoma in different stages, and the predictive value of an autophagy-related lncRNA signature for progression-free survival (PFS). Circular RNAs are a unique class of non-coding RNAs characterized by their covalently closed-loop structure. Circular RNAs regulate gene expression through several mechanisms, such as miRNA sponges, protein scaffolding, transcriptional regulation, and translation into proteins. Wu et al. provided a brief review of some recently discovered carcinogenic and tumor-suppressive circRNAs in neuroblastoma and their working mechanisms with a focus on miRNA sponges. ^18^F-FDG PET/CT metabolic parameters and the presence of *MYCN* oncogene amplification or specific segmental chromosomal aberrations (e.g., 11q deletion, 1p deletion, and 17q gain) can contribute to prognosis assessment in neuroblastoma. Ren et al. attempted to investigate the relationship between these factors, as well as clinicopathological factors and laboratory test parameters. Interestingly, it was shown that lactate dehydrogenase (LDH), the maximal length of the lesion in the axial image (LEGmax), tumor volume (MTV), and total lesion glycolysis (TLG) may serve as effective predictors for *MYCN* oncogene and chromosome 1p36 status in pediatric neuroblastoma and ganglioneuroblastoma. The rapid evolution of gene sequencing and bioinformatics, along with the emergence of relevant tumor databases, opens up new possibilities for deciphering the molecular mechanisms of pediatric cancer and for developing precise drug therapies. Mitochondria are a key target in cancer therapy due to their role in altered energy production in malignant cells. Chai et al. used the machine learning method to identify potential therapeutic targets and prognostic biomarkers from mitochondria-associated proteins (MAPs) in pediatric neuroblastoma, by utilizing cell line-based bulk RNA-seq data, primary neuroblastoma tissue-based bulk RNA-seq data, single-cell RNA-seq (scRNA-seq) dataset of neuroblastoma tissues, *MAP* gene dependency of cell viability dataset, and genome-wide CRISPR screens in primary human T cells. This research broadens the role of *MAP* genes in enhancing neuroblastoma tumor stratification, prognosis prediction, and the development of targeted drugs. Stage “MS” (metastatic special) neuroblastoma 4S, also known as “stage 4 special (4S)”, is characterized by localized primary tumors with limited metastasis. Unlike standard stage 4 neuroblastoma, which is more widespread and aggressive, stage MS may regress spontaneously and have a favorable prognosis. Through bioinformatics analysis, Wu and Zhang compared DEGs between stage M and stage MS neuroblastoma regarding regulatory cell death (RCD), such as apoptosis, autophagy, and ferroptosis. Five genes, *BIRC5*, *SLCO4A1*, *POPDC3*, *HK2*, and *TF*, showed great promise in predicting the prognosis of neuroblastoma. Consistently, Wang et al. also provided evidence of association between RCD and neuroblastoma prognosis. Based on machine learning and relevant tumor databases, they developed risk classifiers consisting of four autophagy-related lncRNAs, which could facilitate the prediction of progression-free survival (PFS) in neuroblastoma.

Additionally, studies on pediatric pilomyxoid and pilocytic astrocytomas, as well as genetic variants in m5C modification core genes in acute lymphoblastic leukemia (ALL), add to the growing body of literature aiming to refine molecular classification and risk stratification in pediatric oncology. The most common pediatric brain tumor, pilocytic astrocytoma (PA), generally grows slowly with favorable outcomes, but its subtype, pilomyxoid astrocytoma (PMA), has a more aggressive course and distinct histology. A genome-wide copy number screening was conducted in one of the largest Saudi cohorts of pediatric patients with PMA and PA (AlShail et al.). Overall, 41 copy number aberrations (CNAs) (34 gains, 7 losses) were detected across all patients, as well as the presence of the KIAA1549-BRAF fusion gene in over 88% of cases (89% in PMA, 80% in PA). Moreover, other genomic CNAs were identified in 12 patients. Pathway analysis showed alterations in retinoic acid-mediated apoptosis, MAPK signaling, and key genes linked to tumor growth, including *BRAF*, *TP53*, and *SOX4*. Genomic sequencing analyses suggest that pediatric cancers typically present with fewer somatic mutations and a higher occurrence of germline alterations in cancer susceptibility genes (5). Genetic predisposition plays a significant role in this patient population, contributing to approximately 10% of cases (6–8). A study on genetic variants in m5C modification core genes in ALL revealed that *NOL1* rs3764909 and *NSUN4* rs10252 polymorphisms were significantly associated with increased susceptibility to pediatric ALL (Wang et al.).

In conclusion, the articles on this Research Topic demonstrate the impacts of molecular diagnostics on early detection, personalized treatment, and improved patient outcomes with the most recent evidence, and they contribute valuable knowledge to this field. Emerging technologies such as multi-omics approaches, AI-driven diagnostics, and targeted genetic testing are leading to more precise and individualized interventions.
